# Production of Galactooligosaccharides Using β-Galactosidase Immobilized on Chitosan-Coated Magnetic Nanoparticles with Tris(hydroxymethyl)phosphine as an Optional Coupling Agent

**DOI:** 10.3390/ijms160612499

**Published:** 2015-06-03

**Authors:** Su-Ching Chen, Kow-Jen Duan

**Affiliations:** Department of Bioengineering, Tatung University, Taipei 104, Taiwan; E-Mail: monica94707@yahoo.com.tw

**Keywords:** chitosan, enzyme immobilization, Fe_3_O_4_ nanoparticles, β-galactosidase, galactooligosaccharides, THP

## Abstract

β-Galactosidase was immobilized on chitosan-coated magnetic Fe_3_O_4_ nanoparticles and was used to produce galactooligosaccharides (GOS) from lactose. Immobilized enzyme was prepared with or without the coupling agent, tris(hydroxymethyl)phosphine (THP). The two immobilized systems and the free enzyme achieved their maximum activity at pH 6.0 with an optimal temperature of 50 °C. The immobilized enzymes showed higher activities at a wider range of temperatures and pH. Furthermore, the immobilized enzyme coupled with THP showed higher thermal stability than that without THP. However, activity retention of batchwise reactions was similar for both immobilized systems. All the three enzyme systems produced GOS compound with similar concentration profiles, with a maximum GOS yield of 50.5% from 36% (w·v^−1^) lactose on a dry weight basis. The chitosan-coated magnetic Fe_3_O_4_ nanoparticles can be regenerated using a desorption/re-adsorption process described in this study.

## 1. Introduction

Galactooligosaccharides (GOS), which can stimulate the proliferation of *Bifidobacteria* and *Lactobacilli* in the intestine, are important food additives for health promotion. GOS can be produced using β-galactosidase (EC 3.2.1.23) to catalyze the hydrolysis of lactose and the transgalactosylation of a galactoside to an acceptor. The acceptor can be a monosaccharide such as glucose or galactose, or a disaccharide, mainly lactose [1]. β-Galactosidases can be produced from various microbial sources [2,3], such as *Aspergillus oryzae* [4], *Bacillus* sp. [5], *Escherichia coli* [6], *Kluyveromyces lactis* [7], *Kluyveromyces fragilis* [8], *Lactobacillus reuteri* [9], *Thermus aquaticus* YT-1 [10], and *Thermotoga maritima* [11].

Enzyme immobilization technology provides lower production costs, higher efficiency, better enzyme reusability, and improved enzyme stability than the free enzyme technology. Various methods for the immobilization of β-galactosidase have been developed, such as physical adsorption [12,13], entrapment [14], and covalent binding [15,16]. However, due to the mass transfer resistance of particle carriers, immobilization of the enzyme often results in a 20%–30% reduction in GOS yield from lactose [17,18,19].

The use of magnetic nanoparticles as a support for enzyme immobilization has several advantages: (1) a higher specific surface area permitting the binding of a larger amount of enzymes; (2) the mass transfer resistance being relatively low; and (3) easy and selective separation of the immobilized enzyme from a reaction mixture by the application of a magnetic field [20,21]. Magnetic nanoparticles have been used for the immobilization of many enzymes, such as lipase [22], chitosanase [23], d-amino acid oxidase [24], β-galactosidase [25], and β-fructofuranosidase [26].

The binding of enzyme to magnetic nanoparticles is commonly accomplished through the coupling agent, or surface coating using polymers such as chitosan, alginate, carrageen. Chitosan has a polycationic character containing cationic NH_3_^+^ groups. Many enzymes that have polyanionic characteristic are immobilized on chitosan by physical adsorption.

Chitosan has been widely used as a carrier of immobilized enzymes due to its abundant supply, low cost, and non-toxicity [27]. The coupling agent most often used between enzymes and chitosan is glutaraldehyde. As a solid support for enzyme immobilization, chitosan can be prepared to a porous spherical particles (Chitoperal™, Fuji Spinning Co., Ltd., Tokyo, Japan), micro- and nanosized particles [28,29]. Enzymes can also be immobilized onto the surface of chitosan beads via a coupling agent such as glutaraldehyde. However, glutaraldehyde is toxic and the C=N bond in the coupling reaction is prone to hydrolysis [30], which reduces the stability of the immobilized enzyme, especially when the immobilized enzyme is used at a high temperature. Alternatively, an ionotropic gelation agent, such as tris(hydroxymethyl)phosphine (THP) [31], can be used as a coupling agent. THP is synthesized through the treatment of tetrakis(hydroxylmethyl)phosphonium chloride (THPC) with a KOH base. THP is a water-soluble substance and can react with chitosan to form a P–CH_2_–N linkage at room temperature. This P-CH_2_-N linkage has improved hydrolytic resistance for covalent immobilization of enzymes.

The magnetic Fe_3_O_4_ nanoparticles of this investigation were prepared using the procedures of previous report [26]. The diameter of the nanoparticles was approximately 4–6 nm by using TEM [26]. Typical diameter of the magnetic Fe_3_O_4_ nanoparticles prepared using a chemical coprecipitation range from 5 to 20 nm [32,33]. The chitosan nanoparticles can be prepared by ionotropic gelation using sodium sulfate or pentasodium tripolyphosphate (TPP) as the gelation agent. The diameter of the nanoparticles is several hundred nm which is much larger than the magnetic Fe_3_O_4_ nanoparticles [28,29].

In this study, chitosan-treated Fe_3_O_4_ (Fe_3_O_4_-CS) nanoparticles were used as a solid support to immobilize β-galactosidase. The chitosan on the nanoparticles was physically cross-linked by ionic gelation using TPP [34]. β-galactosidase was then directly linked to these Fe_3_O_4_-CS nanoparticles, or THP was used as a cross-linking agent between the enzyme and the Fe_3_O_4_-CS nanoparticles (Fe_3_O_4_-CS-THP). We then compared various properties of the two immobilized and the free enzyme systems.

## 2. Results and Discussion

### 2.1. Enzyme Immobilization

**Figure 1:**
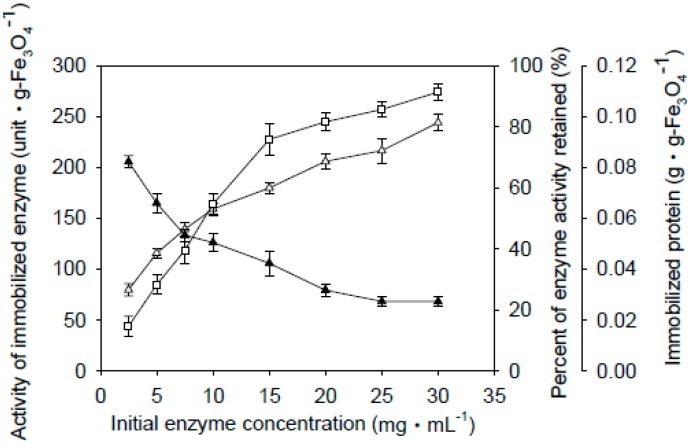
Adsorption equilibria of Fe_3_O_4_-CS-THP-immobilized β-galactosidase. 0.5 g of Fe_3_O_4_-CS-THP was added to 4 mL of β-galactosidase solution at various concentrations, and enzyme immobilization was allowed to proceed with mild shaking at 25 °C for 2 h. The particles were then collected with a permanent magnet and washed with distilled water thrice. The resulting immobilized enzyme was stored in the 0.1 M sodium acetate buffer (pH 6.0) at 4 °C. The data are averaged from three samples. (Δ) Activity of immobilized enzyme; (▲) Percent of enzyme activity retained; (□) Immobilized protein per gram of Fe_3_O_4_-CS-THP nanoparticles.

**Figure 2:**
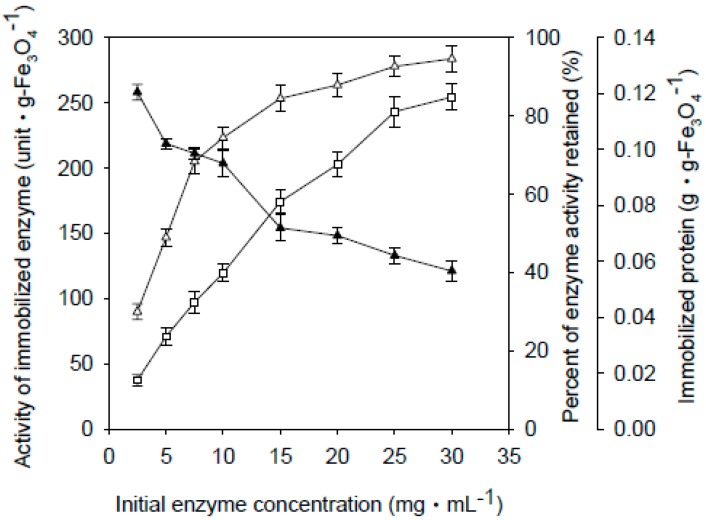
Adsorption equilibria of Fe_3_O_4_-CS-immobilized β-galactosidase. Experimental details are the same as that of Figure 1. The data are averaged from three samples. (Δ) Activity of immobilized enzyme; (▲) Percent of enzyme activity retained; (□) Immobilized protein per gram of Fe_3_O_4_-CS nanoparticles.

Fe_3_O_4_-CS-THP and Fe_3_O_4_-CS nanoparticles were used for enzyme immobilization at various enzyme concentrations. Figure 1 and Figure 2 show the absorption equilibria for different initial enzyme concentrations *versus* the activities of the immobilized enzyme, and the percentage of the adsorbed enzyme activity retained on the Fe_3_O_4_-CS-THP and Fe_3_O_4_-CS nanoparticles, respectively. Interestingly, the Fe_3_O_4_-CS nanoparticles showed better enzyme activity retention than the Fe_3_O_4_-CS-THP nanoparticles. The enzyme activity retained after immobilization ranged from 70% to 23% for the Fe_3_O_4_-CS-THP nanoparticles (Figure 1), and from 85% to 40% for the Fe_3_O_4_-CS nanoparticles (Figure 2). For subsequent experiments, the immobilized enzyme was prepared with an initial enzyme concentration of 20 mg·mL^−1^ for Fe_3_O_4_-CS-THP and 10 mg·mL^−1^ for Fe_3_O_4_-CS.

### 2.2. Effect of Temperature and pH on Enzyme Activity

**Figure 3:**
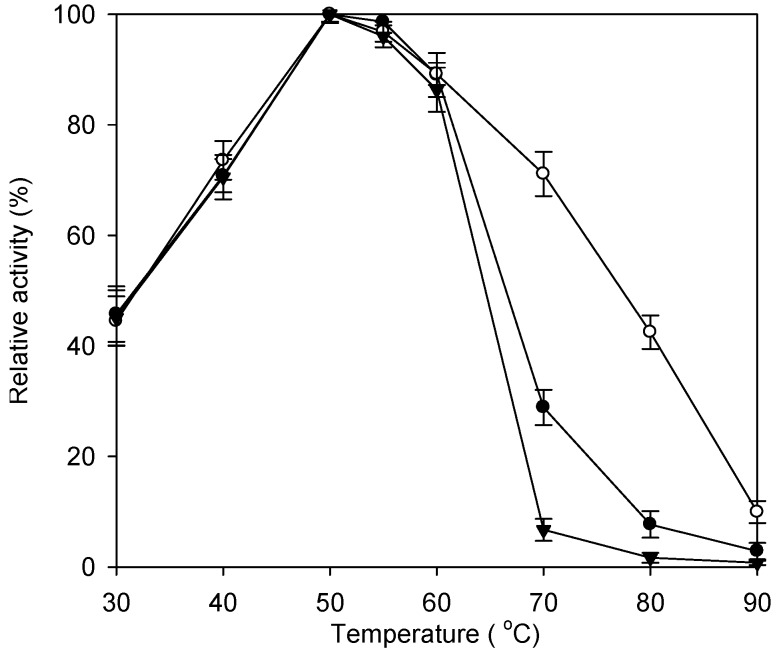
Effect of temperature on the enzyme activity at pH 6.0. Fe_3_O_4_-CS-immobilized enzyme (0.2 g), Fe_3_O_4_-CS-THP-immobilized enzyme (0.2 g) or the free enzyme (1 mL) was added to 100 mL of 5% (w·v^−1^) lactose at pH 6.0 in a 500 mL Erlenmeyer flask and incubated at various temperatures from 20 to 90 °C in an orbital shaker bath at 200 rpm for 10 min. The relative activity was determined by measuring the production of glucose. The data are averaged from three samples. (●) Fe_3_O_4_-CS-immobilized enzyme; (○) Fe_3_O_4_-CS-THP-immobilized enzyme; (▼) free enzyme.

The optimal temperature for the two immobilized enzyme systems and the free enzyme was 50 °C (Figure 3). Immobilized enzymes were more stable than the free enzyme at temperatures higher than 60 °C. In particular, the Fe_3_O_4_-CS-THP-immobilized enzyme showed greatly increased thermal stability at 70 and 80 °C. For example, at 70 °C the Fe_3_O_4_-CS-THP-immobilized enzyme retained 70% of its maximum activity, while the Fe_3_O_4_-CS-immobilized enzyme and the free enzyme retained only 26% and 8% of their activities, respectively. Immobilization restricts flexibility, making enzymes more resistant to unfolding and denaturation at higher temperatures. Indeed, the number of covalent bonds attaching an enzyme to a solid support is correlated with a quantifiable increase in thermal stability, as shown using immobilized trypsin [35]. It is noted that the experiment for the data of Figure 3 was proceeded only 10 min. Cheng *et al.* reported the same free enzyme remained 40% of its initial activity after incubation at 55 °C for 2 days [5]. In order to improve a long term thermal stability, the incubator was set at 45 °C in the subsequent experiment.

**Figure 4:**
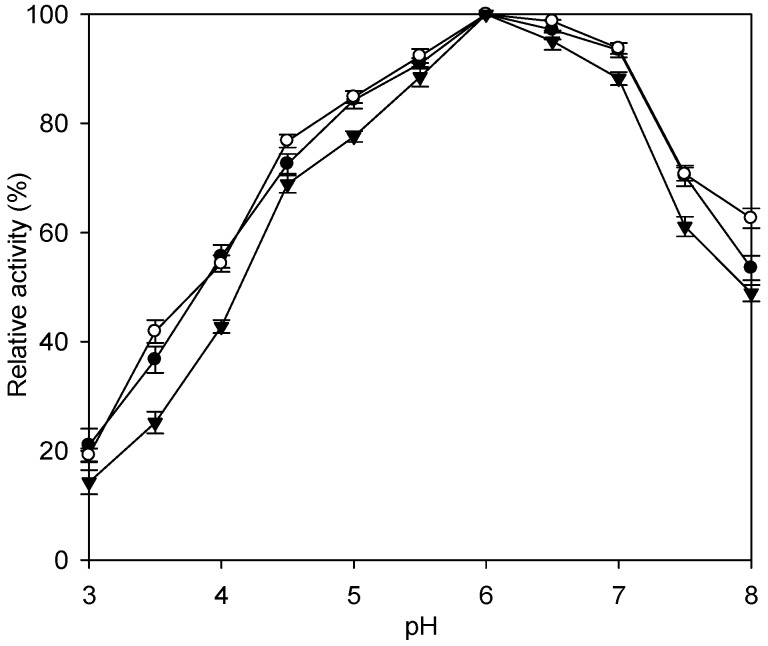
Effect of pH on the relative enzyme activity at 50 °C. Fe_3_O_4_-CS-immobilized enzyme (0.2 g), Fe_3_O_4_-CS-THP-immobilized enzyme (0.2 g) or the free enzyme (1 mL) was added to 100 mL of 5% (w·v^−1^) lactose at 50 °C in a 500 mL Erlenmeyer flask and incubated at various pH from 3.0 to 8.0 in an orbital shaker bath at 200 rpm for 10 min. The relative activity was determined by measuring the production of glucose. The buffer used in this set of reactions were 0.1 M sodium acetate buffer (pH 3.0~5.6) and 0.1 M potassium dihydrogen orthophosphate (pH 5.8~8.0). The data are averaged from three samples. (●) Fe_3_O_4_-CS-immobilized enzyme; (○) Fe_3_O_4_-CS-THP-immobilized enzyme; (▼) free enzyme.

The results of relative activity from the immobilized and free enzymes corresponding to pH changes are shown in Figure 4. The activities of Fe_3_O_4_-CS-THP, Fe_3_O_4_-CS-immobilized enzyme and the free enzymes were maximally achieved at pH 6.0. This result suggests that there was no apparent charge in the immobilization at the active site of the enzyme after coupling with THP. Both the immobilized enzymes show a little improvement of relative pH activity at pH 3.0 to 8.0.

### 2.3. Long-Term Thermal Stability of the Immobilized Enzyme

**Figure 5:**
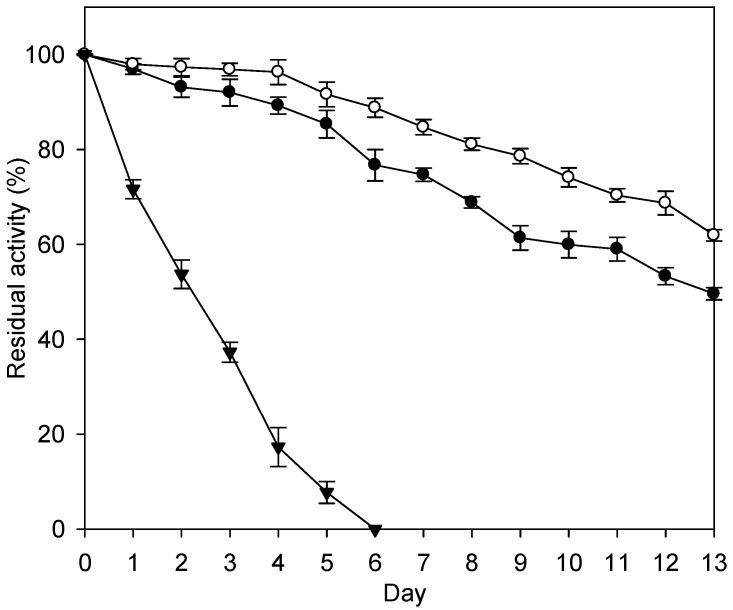
Long-term thermal stability of the enzymes at 45 °C. The data are averaged from three samples. (●) Fe_3_O_4_-CS-immobilized enzyme; (○) Fe_3_O_4_-CS-THP-immobilized enzyme; (▼) free enzyme.

After incubation at 45 °C for 14 days, the residual activities of the Fe_3_O_4_-CS-THP- and the Fe_3_O_4_-CS-immobilized enzymes were 62% and 50%, respectively (Figure 5). The free enzyme showed a complete loss of activity after 7 days. In the Fe_3_O_4_-CS-THP immobilization system, β-galactosidase was covalently linked to the nanoparticles, while in the Fe_3_O_4_-CS system, the enzyme was immobilized to the nanoparticles by ionic force. Covalent bonding may have further restricted the flexibility of the enzyme, rendering the enzyme more resistant to unfolding or denaturation by heat. Cheng *et al.* reported that the same β-galactosidase immobilized on the porous chitosan beads (Chitopearl™) remained 75% residual activities after incubated at 55 °C for 14 days [5]. The results showed thermal stability of β-galactosidase immobilized on the porous chitosan beads was better than that on the nanoparticles of this investigation. Similar findings were obtained by Klein *et al.* where β-galactosidase (Maxilact LX 5000) was covalently bonding on the chitosan beads [28]. The macroparticles (2 mm) showed better thermal stability than that of the nanoparticles (410 nm) [28].

**Figure 6:**
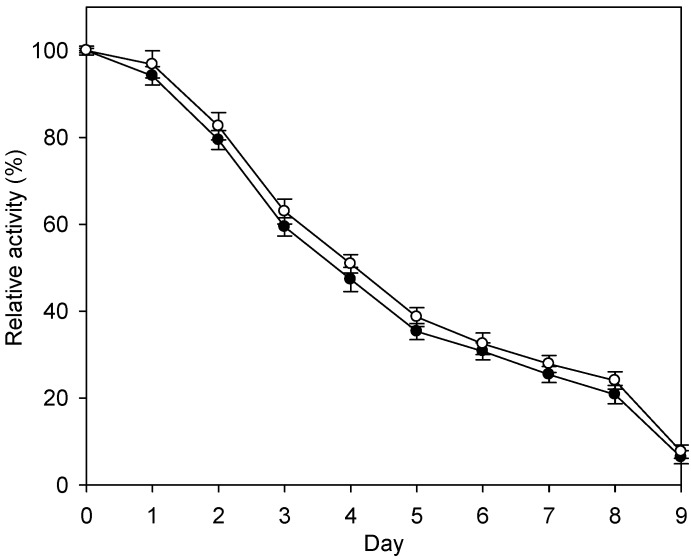
Batchwise reactions of immobilized enzyme at 45 °C. Fe_3_O_4_-CS- or Fe_3_O_4_-CS-THP-immobilized enzymes (0.5 g) was added to 50 mL of 36% (w·v^−1^) lactose at pH 6.0 in a 500 mL Erlenmeyer flask. The solution was incubated 45 °C on an orbital shaker bath of 200 rpm and the lactose solution was replaced every 24 h. The immobilized enzyme was recovered everyday by applying a magnetic field and the residual activity was measured by the standard assay. The procedure was repeated for 9 days. The activity at beginning of the experiments was set as 100%. The data are averaged from three samples. (●) Fe_3_O_4_-CS-immobilized enzyme; (○) Fe_3_O_4_-CS-THP-immobilized enzyme.

In batchwise reactions, enzymes may be detached from the surface of the nanoparticles while being shaken in the flask due to abrasion by the shear forces of the substrate solution. The detached enzyme would be lost when the nanoparticles were retrieved and washed and a new batch of substrate solution added. Therefore, it is important to compare the enzyme retention abilities of the immobilization systems. As shown in Figure 6, in batchwise reactions, the residual activities of the Fe_3_O_4_-CS-THP- and the Fe_3_O_4_-CS-immobilized enzymes were 50.9% and 47.3%, respectively, after incubation at 45 °C for 4 days (Figure 6). Thus, the two systems showed similar enzyme retention abilities. 

### 2.4. GOS Production Using the Free and Immobilized Enzymes

**Figure 7:**
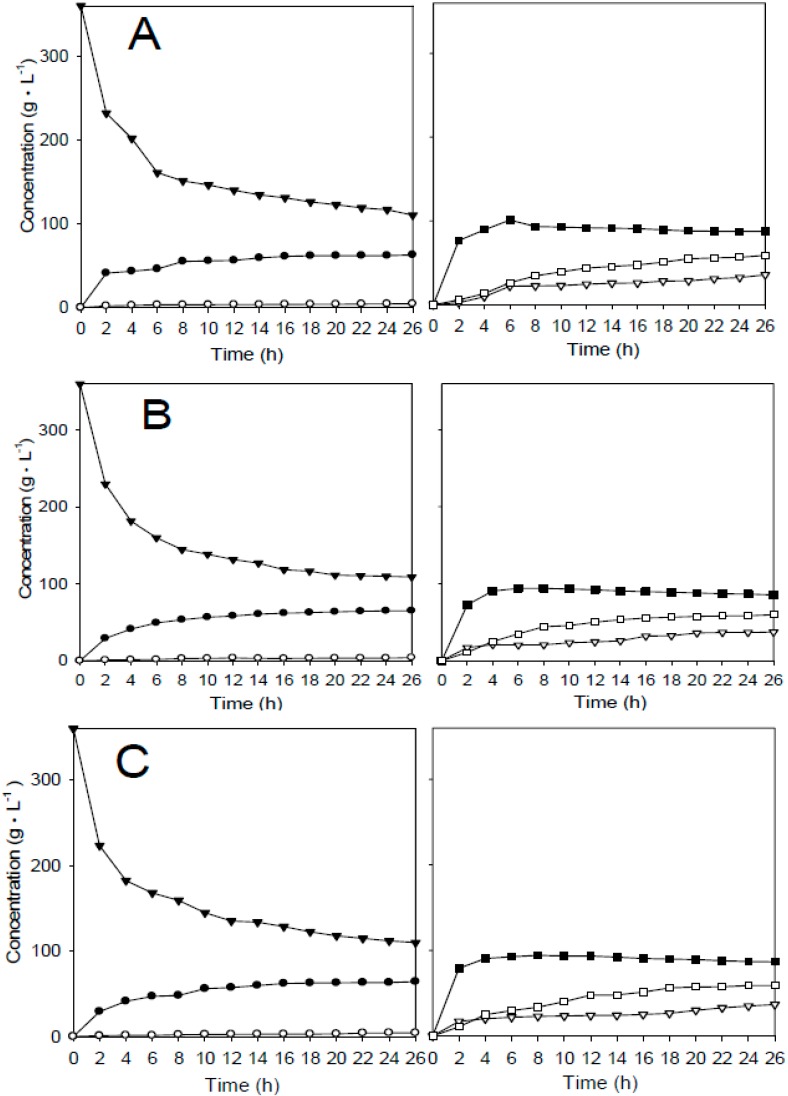
Time-course analysis of various sugars produced. (**A**) free enzyme; (**B**) Fe_3_O_4_-CS-immobilized; and (**C**) Fe_3_O_4_-CS-THP-immobilized enzyme. The reaction was carried out at 45 °C at pH 6.0 with a 36% lactose solution (w·v^−1^). 3.5 units of enzyme were added per gram of lactose. (▼) lactose; (●) glucose; (○) galactose; (▽) disaccharide; (■) trisaccharide; (□) tetrasaccharide.

The concentration profiles of glucose, galactose, disaccharides, trisaccharides, and tetrasaccharides were determined in time-course experiments for the free and immobilized enzymes. The results showed that the three systems have similar product profiles (Figure 7). Trisaccharide is the first transgalactosyl product derived from lactose. Tetrasaccharide is the product of transgalactosylating reaction of a galactoside on the trisaccharide. Two disaccharides were produced by transgalactosylating of galactoside on glucose, probably β-d-Gal-(1→3)-d-Glc and β-d-Gal-(1→6)-d-Glc [1].

**Figure 8:**
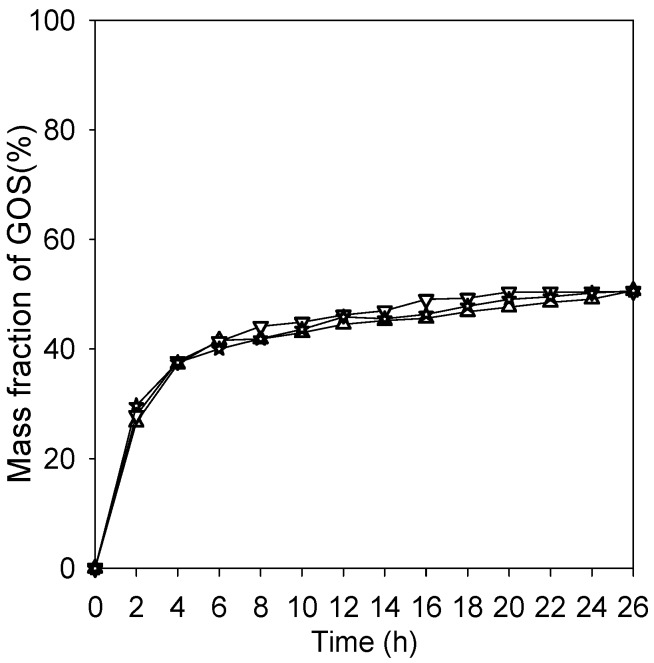
Total GOS production for the three enzyme systems. (Δ) total GOS of free enzyme; (▽) total GOS of Fe_3_O_4_-CS-immobilized enzyme; (☆) total GOS of Fe_3_O_4_-CS-THP-immobilized enzyme.

When the amounts of di-, tri-, and tetrasaccharides were combined to calculate total GOS produced, the results showed that the immobilized and free enzymes all had a maximum GOS yield of 50.5% on a dry weight basis from 36% lactose (*w/v*) (Figure 8). It should be noted that only a 41% yield of GOS was obtained when β-galactosidase was immobilized on porous chitosan beads, because diffusion resistance generated reduced lactose concentrations on the surface of the beads [5]. Under a lower lactose concentration, galactoside is more prone to bind with H_2_O and therefore results a lower yield of GOS from lactose. The nanoparticles take advantage of their high surface area to immobilize sufficient amount of enzyme. Furthermore, the mass transfer resistance of lactose from bulk solution to surface of the nanoparticles is relatively low. It is not surprising that the immobilized enzyme systems performed almost the same reaction characteristics as the free enzyme system.

### 2.5. Reusability of Nanoparticles for Recoupling

**Figure 9:**
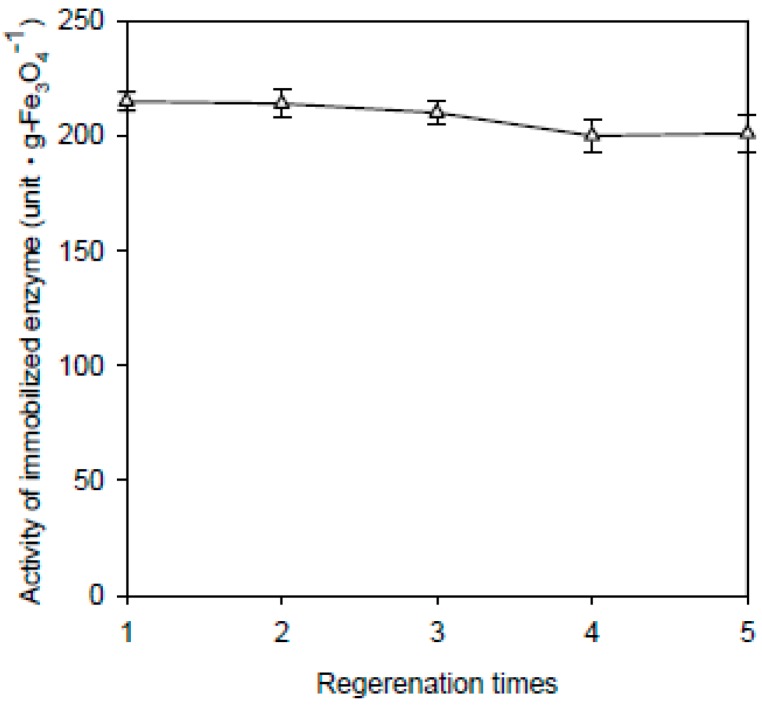
Regeneration of nanoparticles for recoupling. The data are averaged from three samples. (Δ) Fe_3_O_4_-CS-immobilized enzyme.

Immobilized enzymes can lose their activity after several uses. Therefore, for practical applications, the solid support should have good desorption/re-adsorption properties so they can be reused. For regeneration, the Fe_3_O_4_-CS nanoparticles were sequentially washed five times with buffer solutions having pH 9 and 4. Then, the stripped nanoparticles were used to adsorb fresh enzymes. This desorption/re-adsorption cycle was repeated five times. The activity of the β-galactosidase from the fifth adsorption/desorption cycle remained at approximately 92% of that of the first immobilization as shown in Figure 9. Similar results have also been observed by Wang *et al.* [36], who showed that when the enzyme glucoamylase was immobilized onto magnetic chitosan, the nanoparticles displayed good desorption/re-adsorption properties.

## 3. Experimental Section

### 3.1. Preparation of Magnetic Nanoparticles

Magnetic Fe_3_O_4_ nanoparticles were prepared using a chemical coprecipitation technique [37]. The chemical reaction can be expressed as follows:

(1) $${\text{FeCl}}_{2}+{2\text{FeCl}}_{3}+8\text{NaOH}\to {\text{Fe}}_{3}{\text{O}}_{4}+8\text{NaCl}+{4\text{H}}_{2}\text{O}$$

Two grams of FeCl_2_·4H_2_O and 5.4 g of FeCl_3_·6H_2_O (Fe^2+^:Fe^3+^ = 1:2) were completely dissolved in 100 mL of distilled water at 70 °C. Next, 30 mL of 10 N NaOH was added to the above solution and stirred vigorously. The reaction was allowed to proceed for 1 h, and the temperature was maintained at 70 °C. Fe_3_O_4_ nanoparticles were recovered using a permanent magnet and rinsed three times with distilled water.

### 3.2. Preparation of Magnetic Fe_3_O_4_-CS and Fe_3_O_4_-CS-THP Nanoparticles

The chitosan solution was prepared by dissolving 1 g of chitosan powder in 100 mL of 1% (v·v^−1^) acetic acid. The mixture was added to 25 mL of 10 mg·mL^−1^ TPP solution. Cross-linking of chitosan and TPP occurred while the mixture was stirred for the subsequent 1 h. Fe_3_O_4_ nanoparticles were then dispersed in the chitosan solution by stirring for 40 min at 25 °C. Next, the nanoparticles were steeped in 80% (v·v^−1^) ethanol. The Fe_3_O_4_-CS nanoparticles were washed three times with distilled water and stored at 4 °C [34].

THP (2.5 mg·mL^−1^) was synthesized from THPC. For Fe_3_O_4_-CS-THP nanoparticle preparation, 1 mL of THP was added to 0.1 g Fe_3_O_4_-CS and gently mixed at 25 °C for 10 min. The particles were then washed three times with deionized water and stored at 4 °C for future use [5].

### 3.3. Enzyme Immobilization

Half a gram of Fe_3_O_4_-CS-THP or Fe_3_O_4_-CS nanoparticles was added to 4 mL of β-galactosidase solution (20 or 10 mg·mL^−1^), and enzyme immobilization was allowed to proceed for 2 h with mild shaking at 25 °C. The particles were then collected with a permanent magnet and washed three times with distilled water. The protein concentration before and after immobilization was measured by the Bradford method using bovine serum albumin as a standard [38]. The resulting immobilized enzyme was stored in the 0.1 M sodium acetate buffer (pH 6.0) at 4 °C.

### 3.4. Assays of β-Galactosidase Activity

Ninety-nine milliliters of 5% (w·v^−1^) lactose in the 0.1 M sodium acetate buffer (pH 6.0) was incubated with 1 mL of free enzyme at 50 °C for 10 min. The reaction was quenched in ice water. The reaction product, glucose, was measured using a glucose sensor (Model YSI 2700, Yellow Spring Industries, Yellow Spring, OH, USA). To assay the immobilized enzyme, 0.2 g of enzyme-conjugated nanoparticles was added to 100 mL of 5% (w·v^−1^) lactose in a 500-mL Erlenmeyer flask and incubated at 50 °C in an orbital shaker bath at 200 rpm for 10 min. One unit of enzyme activity was defined as the amount of enzyme producing 1 μmol of glucose per minute under the above conditions.

### 3.5. High-Performance Liquid Chromatography (HPLC) Analyses

The weight fraction of beneficial GOS composition was analyzed by cooperation of CARBOSep CHO-620 CA column (Transgenomic, Inc., Omaha, NE, USA) and Chromatorex NH_2_ column (Fuji Silysia Chemical Ltd., Kasugai Aichi, Japan). Glucose, galactose, disaccharides, trisaccharides, tetra-saccharides and penta-saccharides can be identified by the CARBOSep CHO-620 CA column. The disaccharides composed of lactose, β-d-Gal-(1→3)-d-glu, and β-d-Gal-(1→6)-d-glu which can be identified by NH_2_-column. The beneficial GOS weight fraction are calculated by summation of the absorption area fraction for trisaccharides, tetra-saccharides and penta-saccharides as well as β-d-Gal-(1→3)-d-glu, and β-d-Gal-(1→6)-d-glu of the disaccharides. The mobile phase for CARBOSep CHO-620 CA column was water at 0.5mL·min^−1^. The detector was a differential refractometer held at 90 °C. The mobile phase for Chromatorex NH_2_ column was water–acetonitrile (25:75, v·v^−1^) at 0.5mL·min^−1^. The detector was a differential refractometer held at 30 °C.

## 4. Conclusions

Our data indicate that both the Fe_3_O_4_-CS and Fe_3_O_4_-CS-THP nanoparticles are suitable carriers for β-galactosidase immobilization for efficient GOS production. The composition and yield of GOS were similar between the immobilized and the free enzyme systems. Using 36% (w·v^−^^1^) lactose, we achieved 50.5% yield GOS in both the immobilized enzyme and free enzyme systems. Immobilized β-galactosidase showed the same or higher activity at a wider range of temperatures and pH than did the free enzyme. Coupling with THP further enhanced the thermal stability of the immobilized enzyme. The Fe_3_O_4_-CS nanoparticles can be regenerated and re-used for a new immobilization.
